# Computer Vision-Based Information Extraction from Electrical Assets: A Systematic Literature Review

**DOI:** 10.3390/s26165009

**Published:** 2026-08-07

**Authors:** Carolina Alvarez-Murillo, Anna Ospina-Bedoya, John W. Branch-Bedoya, Germán Darío Zapata Madrigal, Rodolfo García Sierra

**Affiliations:** Facultad de Minas, Universidad Nacional de Colombia, Medellín 050041, Colombia; caalvarezmu@unal.edu.co (C.A.-M.); jwbranch@unal.edu.co (J.W.B.-B.); gdzapata@unal.edu.co (G.D.Z.M.); rodolfo.garcia@enel.com (R.G.S.)

**Keywords:** computer vision, text extraction, electrical equipment, electrical nameplates, systematic literature review, deep learning, OCR, DBNet, YOLO, CRNN

## Abstract

Automated inspection of electrical equipment has become increasingly important as utilities seek to replace inefficient manual processes in infrastructure management. Electrical-equipment nameplates contain critical technical information, but automatically extracting this information remains challenging. Although this topic has attracted growing research interest, the literature has not yet been systematically synthesized. This review addresses three research questions: (1) What real-world challenges affect the inspection and maintenance of electrical equipment, and what research gaps and opportunities remain? (2) Which computer vision and deep learning algorithms and architectures are used to extract text and digits from electrical equipment images? (3) Which metrics, datasets, and evaluation methodologies are used to assess these approaches? We conducted a systematic literature review in accordance with PRISMA 2020. Peer-reviewed English-language studies published between 2020 and 2026 were retrieved from IEEE Xplore, Scopus, ScienceDirect, SpringerLink, the ACM Digital Library, and complementary semantic-search sources; the final searches were conducted in May 2026. The screening and eligibility process reduced 19,760 initial records to 66 primary studies. Owing to substantial heterogeneity in tasks, datasets, and metrics, the evidence was synthesized narratively and in structured tables rather than through meta-analysis. DBNet and CRNN were among the most frequently used architectures for text detection and recognition, respectively, whereas integrated end-to-end systems emerged as a promising research direction. The scarcity of standardized public datasets remains a major barrier to objective comparison and cumulative progress in the field.

## 1. Introduction

The digital transformation of production systems has driven the adoption of artificial intelligence and computer vision technologies in sectors that have historically relied on labor-intensive manual processes. In the electrical sector, thousands of assets, such as transformers, meters, switchgear cabinets, and poles, require periodic inspections to ensure operational safety. The information required for these inspections is recorded on the equipment nameplates of each piece of equipment, which contain manufacturer’s specifications, nominal parameters, and production dates that are essential for maintenance and failure analysis [1].

However, information collection remains largely manual, resulting in long processing times, transcription errors, and the underutilization of the large volume of data generated daily [2,3]. Although the industry has made progress toward automation through machine vision systems, real-world electrical inspection environments impose adverse conditions that no existing standard method fully addresses, thereby requiring solutions specifically tailored to the characteristics of this domain [3,4].

This automation potential extends beyond routine inspection. Digitized nameplate and meter data can feed digital twin models of electrical distribution networks [5], and comparable digital modeling approaches have already been proposed for quality control in electrical cubicle manufacturing [2]. Reliable, automated meter reading has likewise been proposed as a basis for predictive maintenance and anomaly detection in smart metering systems [6], linking the specific task of text extraction to broader goals of grid observability and asset management. Realizing this potential in practice, however, still depends on models that generalize from laboratory conditions to the variability of field deployment—a requirement that is rarely evaluated in the reviewed literature.

Despite the growing number of studies addressing these problems individually, no recent review has consolidated evidence across nameplates, meters, cabinets, datasets, metrics, and real-world inspection constraints for visual information extraction from electrical assets. This gap motivates the present work, whose objective is to provide a Systematic Literature Review of existing approaches to text extraction in this domain. This review is organized as follows: Section 2 describes the methodology used. Section 3 presents the results. Section 4 discusses the limitations of the study and, finally, Section 5 presents the conclusions and future work.

## 2. Methodology

This study was conducted as a Systematic Literature Review (SLR), defined as a structured approach for identifying, evaluating, and interpreting the available scientific evidence addressing a specific research question [7]. The SLR enables the synthesis of existing knowledge on computer vision-based text extraction techniques applied to electrical equipment, ensuring a rigorous and reproducible analysis of the state-of-the-art techniques.

The methodological design followed a replicable and transparent protocol, guided by the Preferred Reporting Items for Systematic Reviews and Meta-Analyses (PRISMA) 2020 statement [8]. In particular, the stages of search, selection, quality assessment, and data extraction were explicitly defined to ensure traceability, minimize bias, and enable the reproducibility of the process. The review strategy included the formulation of research questions, the construction of search strings based on domain-specific keywords, and the application of inclusion and exclusion criteria to an initial set of studies retrieved from multiple databases.

### 2.1. Scope Definition

This systematic literature review aimed to provide an overview of the current research on text, character, and digit extraction algorithms applied to electrical equipment in industrial and power system contexts. In particular, the review focused on identifying computer vision, OCR, machine learning, and deep learning approaches used in this domain; examining the evaluation metrics and methodologies reported in the literature; and identifying the main challenges that affect their performance under real-world operating conditions.

Following Petersen et al.’s guidelines for systematic mapping studies [9], the review scope was defined using an adapted PICO framework to the objectives of this study. This framework was used to structure the review in terms of the target domain, the technological approaches under analysis, the types of comparisons considered, and the outcomes used to assess performance. Accordingly, the population was defined as electrical equipment used in industrial and energy environments. The intervention consisted of computer vision, OCR, machine learning, and deep learning techniques for text, character, and digit extraction. Comparators included traditional image processing and OCR-based methods, as well as recent deep learning architectures, while the outcomes comprised performance metrics, datasets, evaluation methodologies, and reported limitations under real-world inspection conditions.

Based on the defined scope, the following research questions were formulated:

1. **RQ1**: What are the main real-world challenges affecting the inspection and maintenance of electrical assets, including transformers, switchgear panels, poles, and equipment nameplates, and what research gaps and opportunities are identified in this field?
2. **RQ2**: What computer vision and deep learning algorithms and architectures are currently used for text and digit extraction in electrical equipment?
3. **RQ3**: What metrics, datasets, and evaluation methodologies are used to measure the performance of these approaches in recent literature?

### 2.2. Search Strategy

To translate the research questions into operational search terms, the keywords were organized into four conceptual groups: application domain, text extraction task, computer vision and OCR technologies, and field conditions or evaluation metrics. This organization aligned the review scope with the technical terminology used in the literature on automated inspection, text recognition, and electrical equipment monitoring. Based on these groups, complementary Boolean search strings were constructed to balance thematic breadth with technical specificity. The resulting keyword groups and search terms are summarized in Table 1.

The search strings were constructed through progressive combinations of the four conceptual groups. The first two strings had a broader scope and were intended to retrieve studies related either to the electrical domain and text extraction tasks or to computer vision and deep learning technologies applied to inspection problems. The third string integrated the application domain, the text extraction task, and the technological approaches in order to identify studies more directly aligned with the focus of this review. Finally, the fourth search string incorporated evaluation terms and field-condition descriptors to retrieve studies reporting performance metrics, datasets, operational constraints, or experimental evidence under real-world inspection conditions.

Searches were performed primarily in the title, abstract, and keyword fields, according to the search fields available in each database or search engine. When a platform imposed restrictions on syntax, query length, or the maximum number of terms, the strings were adapted without altering the underlying logic of combining terms across conceptual groups. This allowed the review to maintain a consistent search strategy across sources while accounting for the specific technical constraints of each academic search engine.

### 2.3. Information Sources

The formal search was conducted across five scientific databases recognized for their coverage of engineering, computer science, artificial intelligence, and industrial applications: IEEE Xplore, Scopus, ACM Digital Library, ScienceDirect, and SpringerLink. In addition, Google Scholar and Semantic Scholar were used as exploratory academic search engines to broaden the search and identify potentially relevant studies outside the primary database set. All sources were searched using the search strings defined in Table 2. Searches were limited to English-language documents published between 2020 and 2026, and were conducted in titles, abstracts, and keywords whenever supported by the platform.

The formal search was restricted to the sources listed in Table 3, which were selected to ensure consistent search procedures, metadata traceability, and reproducible record management.

The five formal sources enabled the retrieval of studies published in journals, conference proceedings, and academic repositories, while the exploratory search engines supported the complementary retrieval of recent studies related to computer vision, OCR, deep learning, and their applications in electrical equipment or industrial contexts. Google Scholar was used only as an exploratory cross-check source. For each conceptual query, the first five pages of results were manually inspected to identify potentially relevant complementary studies, including early online records or gray literature items not retrieved from the formal sources. However, because Google Scholar results are dynamic, not fully reproducible, and not consistently exportable as a complete record set, these records were not included in the formal initial count.

### 2.4. Inclusion and Exclusion Criteria

To ensure the quality, relevance, and traceability of the included studies, only documents that met previously defined criteria were considered. These criteria ensured that only studies directly related to the automatic extraction of text, characters, or digits from electrical equipment using computer vision, optical character recognition, machine learning, or deep learning techniques.

The 2020–2026 time frame was selected on methodological grounds rather than as an arbitrary cut-off. This period reflects the consolidation of the deep learning techniques that define the current technical landscape of the field: differentiable scene-text detectors such as DBNet [10], transformer-based text recognition [1], and successive generations of YOLO-based object detectors [11,12] were introduced or reached maturity within these years, substantially changing how text and digit extraction in electrical assets is approached. Restricting the search to this window therefore captures the body of literature that is methodologically comparable and operationally relevant to present-day inspection systems, while still including the earliest studies that documented the transition from traditional handcrafted methods to deep learning in this domain [3].

Eligibility and methodological relevance were assessed through a staged screening process rather than through a separate numerical quality score. After the initial cleaning and duplicate removal stage, a deterministic keyword-assisted filter was applied to the available metadata, using four keyword groups aligned with the search strategy: application domain, text extraction task, computer vision or OCR technology, and field conditions or evaluation metrics. In the preliminary pass, records were retained when their keywords matched at least two of these groups. In a subsequent pass, the filter was made more restrictive by requiring at least one application-domain term and matches in at least two groups overall. This keyword-based step was used to reduce and prioritize the candidate set for manual review; it was not treated as a methodological quality score and did not replace reviewer judgment. Final eligibility was determined by reading titles, abstracts, keywords, and, when necessary, the full text, verifying whether each study satisfied the inclusion and exclusion criteria and provided sufficient methodological or empirical evidence to answer at least one research question. The eligibility criteria applied during study selection are summarized in Table 4.

To organize the evidence transparently for synthesis, the included studies were further classified into three tiers according to their proximity to the review’s target domain, as summarized in Table 5. This stratification distinguishes studies that address electrical assets directly from those that provide related industrial or purely methodological evidence, and is used in the results section (Section 3) to assign different weight to the findings according to their relevance to the electrical inspection domain.

### 2.5. Selection and Collection Process

The selection and data collection process was initiated using the search strings defined in Table 2 and applied to the information sources described above. Whenever supported by the platform, searches were limited to titles, abstracts, and keywords, to retrieve studies relevant to the scope of the review and minimizing the inclusion of results outside the scope of the review.

Q1–Q4 correspond to the search-string combinations defined in Table 2. The same conceptual search strategy, publication period, language restriction, and screening criteria were applied across all sources. However, all ScienceDirect searches required query simplification because the platform limited each query to a maximum of eight terms or expressions. Therefore, the ScienceDirect versions of Q1–Q4 were adapted by retaining the most representative terms from each conceptual group while preserving the intent of the original search strategy.

Initially, 17,135 records were identified in the five formal sources included in the review: IEEE Xplore, Scopus, ScienceDirect, SpringerLink, and ACM Digital Library. In addition, 2625 records were retrieved through Semantic Scholar, an exploratory AI-assisted academic search engine used to broaden recall during the initial stage. Therefore, the total initial record count reported in Table 6 includes both the five formal sources and Semantic Scholar, yielding 19,760 records before duplicate removal. Google Scholar was also consulted on 17 May 2026 as an exploratory cross-check source, using the same conceptual query groups defined in Table 7. For each query, the first five result pages were manually inspected to verify the comprehensiveness of the search strategy and identify potentially relevant studies not retrieved from the other sources. However, because Google Scholar does not provide stable, fully reproducible result counts and the inspected raw records were not exported or stored, these results were not incorporated into the initial record count. After duplicate checking and eligibility screening, no additional studies from Google Scholar were included in the final corpus.

The records retrieved from the five formal sources and Semantic Scholar were organized into a review matrix, in which basic bibliographic information for each document was consolidated, including title, authors, year of publication, source, abstract, keywords, and any available link or unique identifier. Subsequently, duplicate documents were removed, reducing the initial set to 5464 unique records. In the first screening phase, the documents were screened based on their titles, abstracts, and keywords. At this stage, studies whose main focus was irrelevant to the review or fell outside its scope of the review were excluded, including studies on general OCR, document recognition, or computer vision without an explicit connection to electrical equipment, energy assets, or comparable industrial contexts. After this preliminary screening, 985 potentially relevant studies remained.

During the second screening phase, the remaining 985 studies were reviewed based on their abstracts and, when necessary, their full text. This stage assessed whether each article addressed at least one of the research questions, whether it implemented or evaluated an applicable computational method, and whether it reported experimental results or quantitative performance metrics. Articles that did not meet these criteria, that did not provide sufficient information to assess their results, or that did not contribute direct evidence for the final synthesis were excluded. As a result, 919 studies were removed during the eligibility phase.

Finally, after applying the staged screening and eligibility assessment described above, including full-text assessment where necessary, a final corpus of 66 primary studies was established. This corpus served as the basis for the comparative analysis and evidence synthesis.

**Figure 1:**
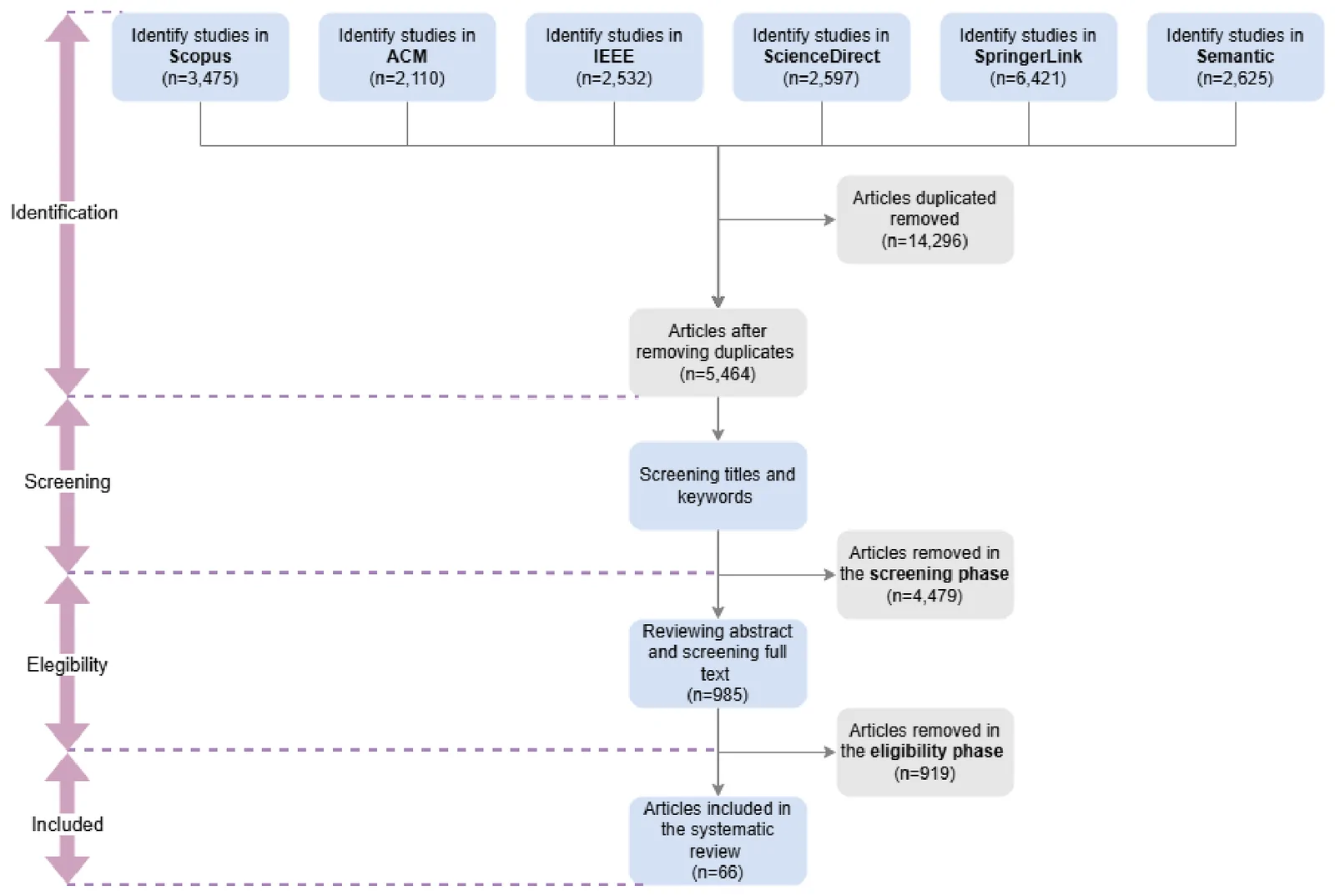
Summary review protocol (PRISMA 2020 flowchart). *Note: Blue boxes show the records retrieved, retained, or assessed, whereas gray boxes show those removed or excluded. Solid arrows trace the flow of records, and the purple guides separate the identification, screening, eligibility, and inclusion stages*.

The complete flow of identification, screening, eligibility, and inclusion is summarized in Figure 1, which was prepared in accordance with the PRISMA 2020 guidelines and provides a transparent framework for reporting the study selection process in systematic reviews [8].

**Figure 2:**
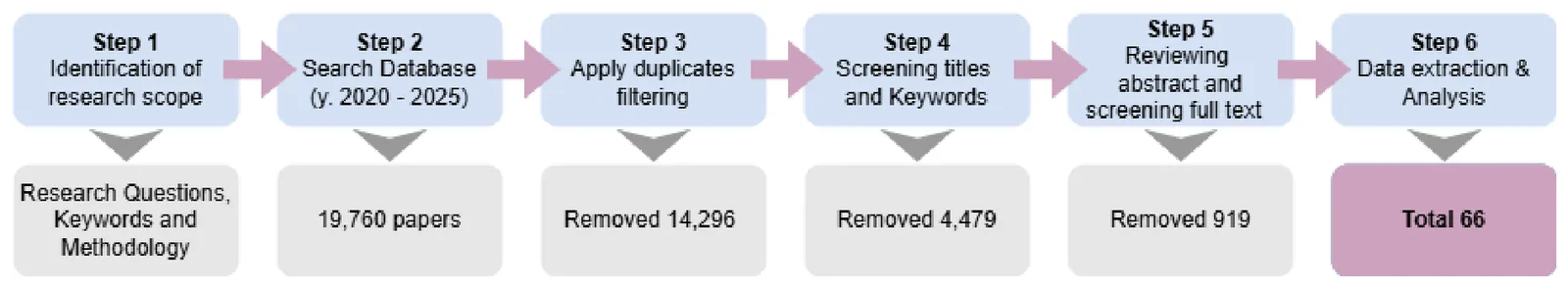
Steps in the systematic review process.

Additionally, Figure 2 summarizes the general stages followed during the systematic review, from defining the scope and constructing the search strings to selecting the primary studies and extracting the data.

**Figure 3:**
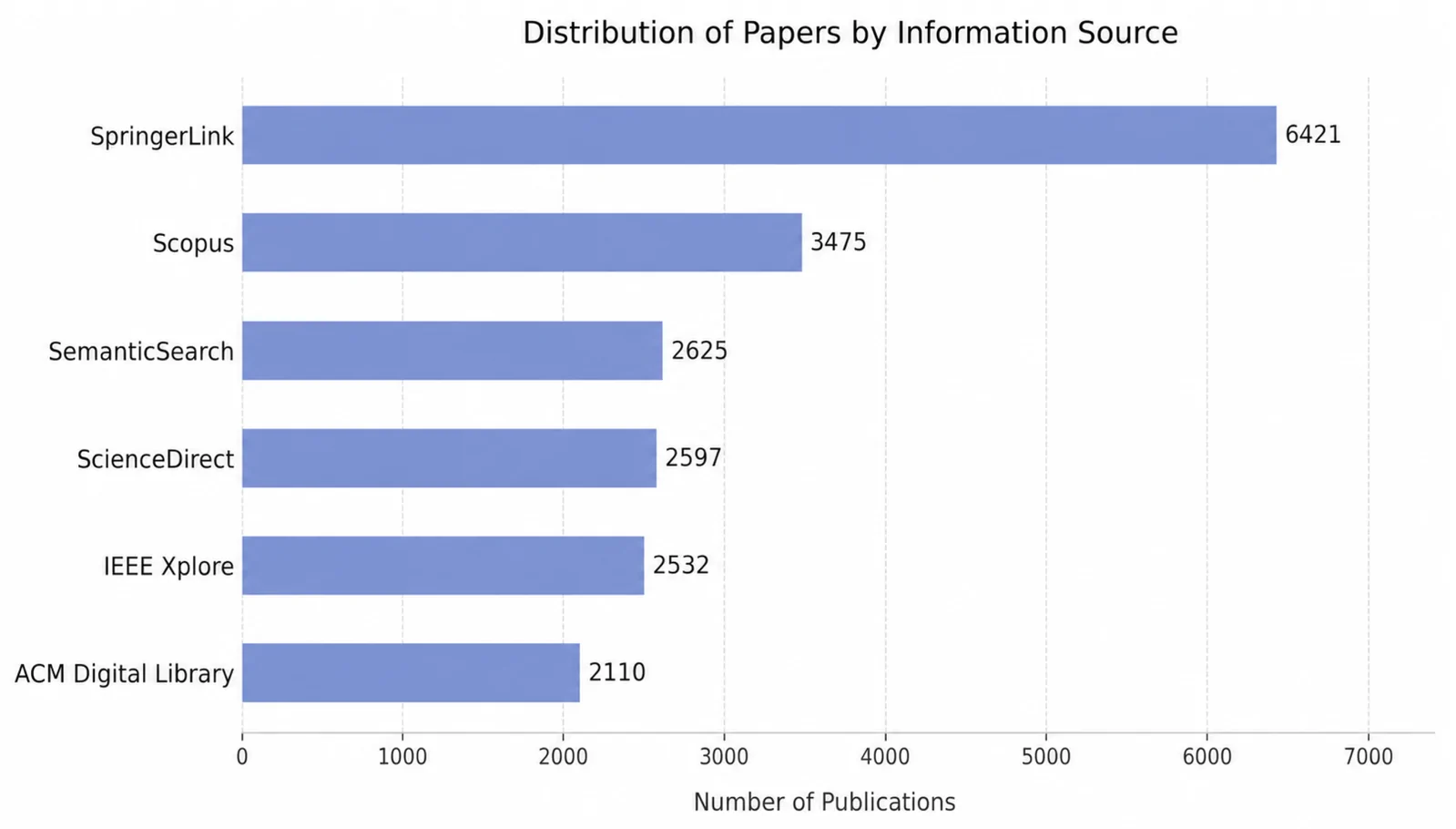
Distribution of articles in the initial corpus by information source.

**Figure 4:**
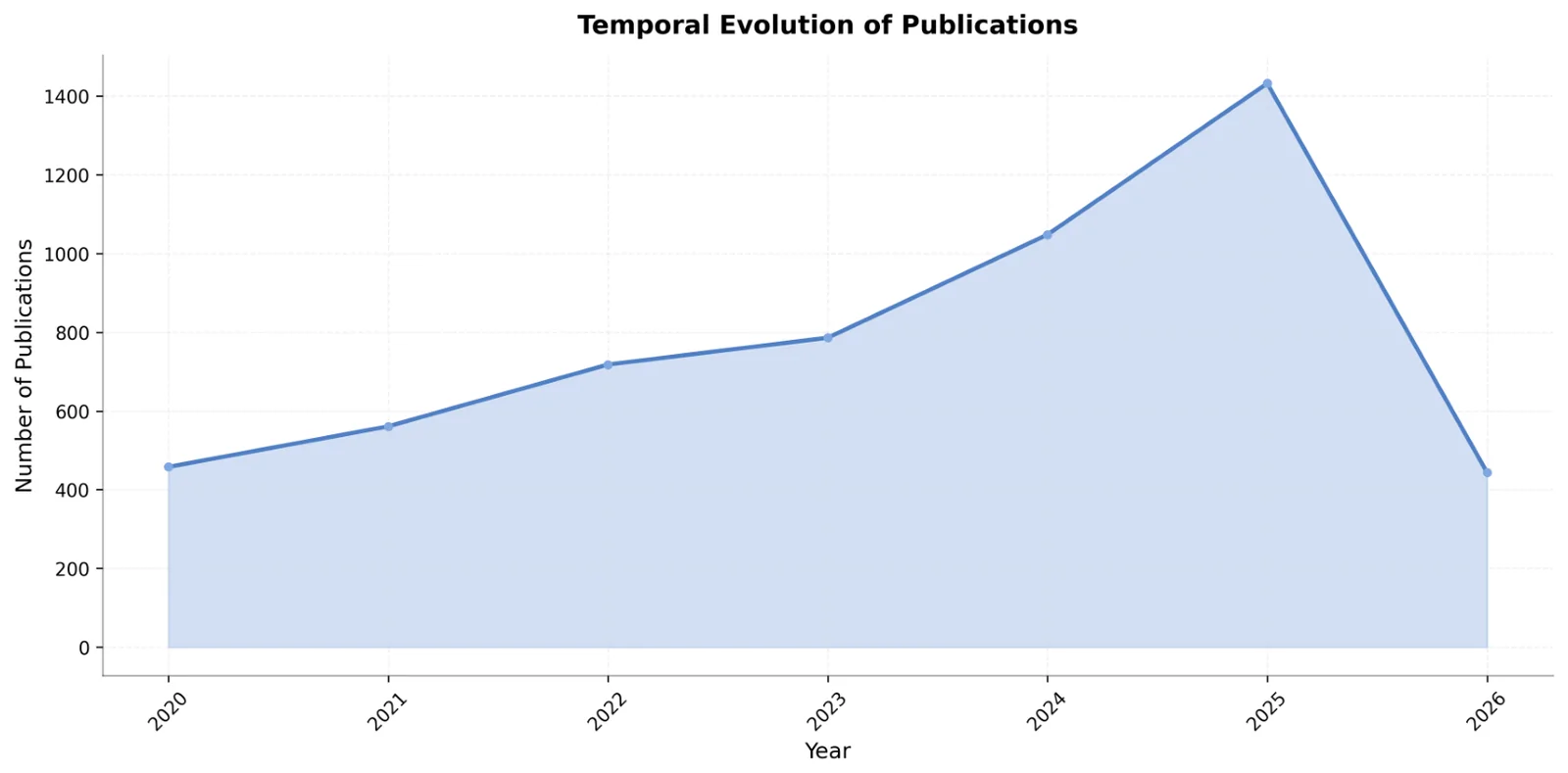
Evolution of the number of publications per year in the initial corpus.

To characterize the retrieved corpus, descriptive figures were generated to illustrate both the distribution across information sources and the temporal evolution of the retrieved records. Figure 3 presents the distribution of articles in the initial corpus by information source, considering the sources consulted during the review process. Meanwhile, Figure 4 shows the evolution of the number of publications by year, highlighting recent trends in research on text extraction from electrical equipment using computer vision and related techniques.

The publication trend shows a sustained increase in publications related to text extraction, OCR, computer vision, and deep learning applications for electrical and industrial equipment between 2020 and 2026. This growth suggests increasing academic interest in the automation of inspection and information extraction tasks in this domain, with a particularly pronounced increase in 2024 and 2025. Although the number of records for 2026 is lower than that of 2025, this value should be interpreted cautiously because the year is still ongoing. Even so, more than 400 publications had already been identified in major academic sources, indicating that research activity in this field remains active and continues to expand.

**Figure 5:**
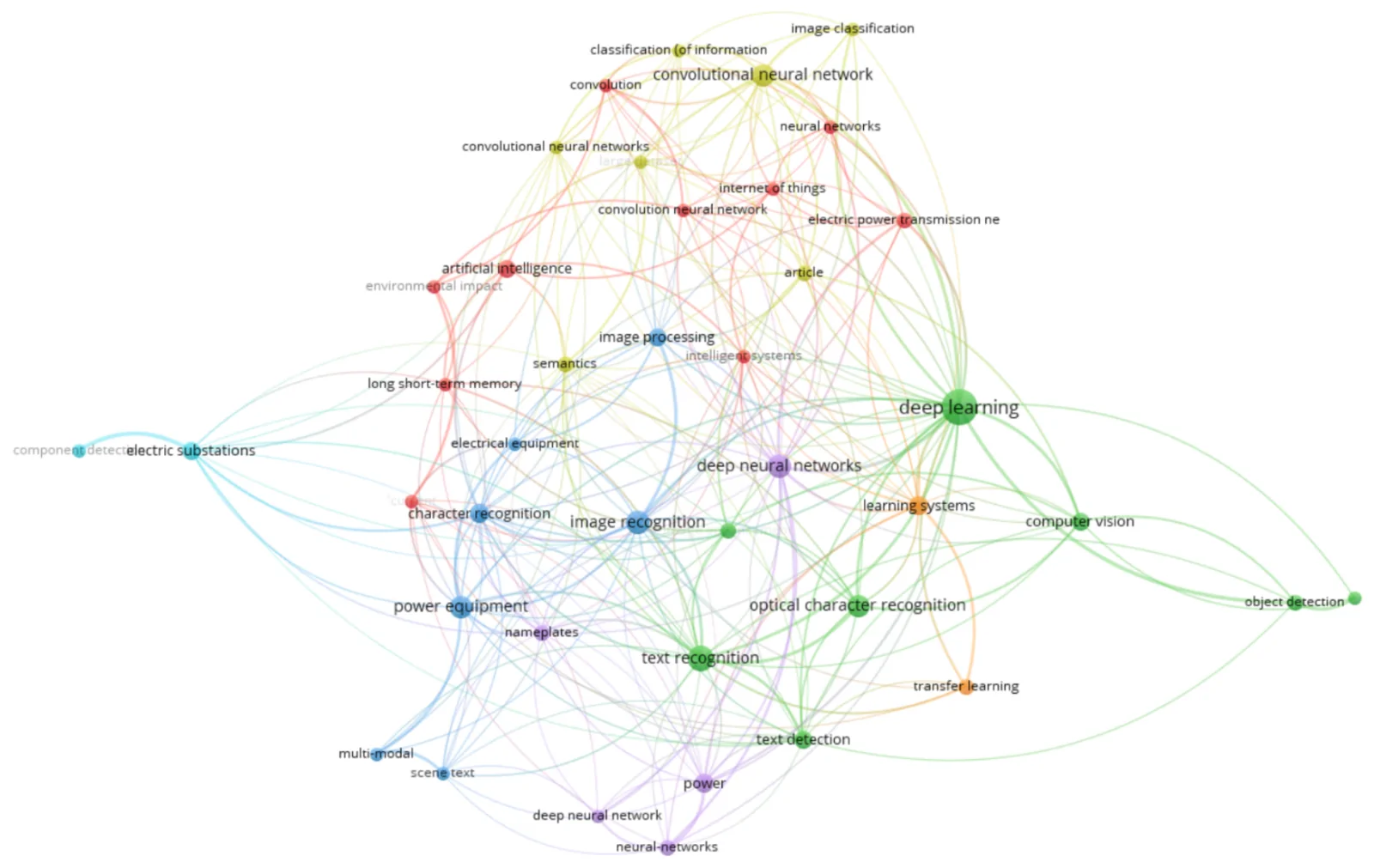
Keyword co-occurrence clusters in the reviewed corpus. *Note: Colors identify different thematic clusters within the keyword co-occurrence network. Although some links and peripheral labels overlap because of the network density*.

In addition to the descriptive analysis by source and publication year, a keyword co-occurrence map was generated to examine the thematic structure of the reviewed corpus. This visualization complements the previous characterization by showing how the main concepts in the literature are grouped and connected. As shown in Figure 5, the most prominent terms are associated with deep learning, convolutional neural networks, optical character recognition, text recognition, image recognition, and power equipment. These relationships indicate that the reviewed literature is concentrated at the intersection of computer vision-based recognition methods and their application to electrical assets, substations, meters, and equipment nameplates.

The map reveals several closely connected thematic clusters. One cluster focuses on deep learning, computer vision, object detection, text recognition, and optical character recognition. This reflects the predominance of neural approaches for automated visual extraction. Another cluster brings together electrical substations, power equipment, electrical equipment, nameplates, image recognition, and character recognition. This reflects the domain-specific orientation of the corpus. A further cluster comprises terms related to convolutional neural networks, image classification, artificial intelligence, the Internet of Things, and intelligent systems suggests that CNN-based architectures remain a dominant technological foundation in the field. Overall, the co-occurrence structure aligns with the scope of this review since the most central terms correspond to the three main dimensions addressed in the research questions: the electrical inspection domain, optical character recognition (OCR) and text extraction tasks, and computer vision and deep learning techniques.

The corpus was dominated by primary empirical and methodological studies. Only one of the included studies corresponded to a prior review, focused specifically on image recognition in power nameplate images [3], confirming the limited availability of broader systematic syntheses across different types of electrical assets. Study selection and data extraction were performed by two reviewers (A.O.B. and C.A.M.). For both tasks, the studies were divided between the two reviewers. Each reviewer’s assignments were then cross-checked by the other, ensuring that every record and every included study was examined independently by both reviewers. Disagreements were resolved through discussion until consensus was reached. During selection, the reviewers screened the titles, abstracts, and keywords of the deduplicated records against the eligibility criteria and subsequently assessed the full text of the potentially relevant reports. Data were extracted into a shared, structured review matrix built in Microsoft Excel, with values recorded directly from the text and tables of each study; bibliographic records from all sources were consolidated in the same matrix, and duplicates were removed manually by matching titles, authors, and DOIs. When a single study was described in more than one report, the most complete report was used as the primary source, and the remaining reports were consulted only to complement missing fields. All screening and extraction were carried out manually. Apart from the deterministic keyword-assisted filtering described above, no machine learning, generative AI, or other automated decision-making tools were used for study selection or data extraction, and study authors were not contacted to confirm eligibility or to obtain data. Because the eligibility criteria restricted inclusion to English-language documents, no translation was required.

### 2.6. Data Items: Outcomes

For each included study, we sought the outcomes used to characterize the performance of computer vision-based text and digit extraction in electrical assets, and outcome data were extracted to assess them. The following outcome domains were defined a priori:

- **Detection performance**: quality of text or object localization, typically reported as precision, recall, F-measure (Hmean), or mean average precision (mAP@0.5).
- **Recognition performance**: accuracy of character or sequence recognition, reported as recognition accuracy and, in some studies, specialized metrics such as the Normalized Average Edit Distance (NAED) and R-Precision.
- **Reading error**: task-specific error measures, such as the relative reading error reported for pointer and digital meters.
- **Preprocessing and restoration quality**: image-quality gains from preprocessing or restoration, reported as Peak Signal-to-Noise Ratio (PSNR) and Structural Similarity Index (SSIM).
- **Computational performance**: operational efficiency, reported as inference latency per frame, training time, or memory footprint.
- **Robustness under field conditions**: performance retention under adverse acquisition conditions such as tilt, varying illumination conditions, blur, and occlusion.

When a study reported several results corresponding to the same outcome domain, the result obtained under the most representative evaluation setting was retained for the synthesis.

### 2.7. Data Items: Study and Context Variables

Beyond outcomes, the following study- and context-level variables were extracted to characterize each study and to enable grouping and comparison: bibliographic identifiers (authors, year, and country); asset type (e.g., nameplate, meter, cabinet, pole, or diagram); task (preprocessing, detection, recognition, end-to-end, or post-processing); algorithm or model architecture; dataset name, size, and availability (public or private); reported evaluation metrics; hardware and experimental environment; validation setting (laboratory, controlled field, real substation, public benchmark, or synthetic data); reported limitations; and an overall quality assessment. When information for a variable was missing or ambiguous in a report, the field was recorded as “not reported” rather than inferred, and this limitation was taken into account when interpreting the corresponding study. Quality assessment was integrated into the eligibility screening described above rather than treated as a separate scoring stage. During full-text assessment, both reviewers verified that each candidate study applied at least one computer vision, OCR, machine learning, or deep learning technique within the scope of the review; reported the results and discussed the advantages and limitations of the approach; when experimental results were presented, specified the metrics used, the reported values, and the dataset employed, including whether it was publicly available or privately collected. Studies not meeting this minimum standard of reporting were excluded during the eligibility phase, and each remaining study was further discussed jointly to determine which research question(s) each study addressed. The outcome of this assessment is reflected in the study- and context-level data items described above.

### 2.8. Synthesis Methods

Because the included studies differ substantially in tasks, asset types, datasets, and evaluation metrics, a quantitative meta-analysis was neither feasible nor appropriate. Pooling effect sizes across such heterogeneous outcomes would not yield interpretable estimates. Consequently, a narrative synthesis supported by structured tables was adopted. Each study was first assigned to the research question (or questions) it addressed and then grouped along five dimensions: the extraction task (preprocessing, detection, recognition, end-to-end, or post-processing), the asset type, the algorithmic family (e.g., DBNet, CRNN, YOLO, or transformer-based models), the dataset used, and the reported evaluation metric. Within each group, results were tabulated and compared qualitatively, with explicit attention to the evaluation setting so that laboratory or benchmark results were not conflated with performance under real-world conditions. The frequency with which each architecture, challenge, dataset, and metric is reported across the corpus in the results as a simple count of contributing studies, providing a transparent and reproducible basis for the qualitative claims.

## 3. Results

### 3.1. Results for Research Question 1

What are the main real-world challenges associated with the inspection and maintenance of electrical equipment such as transformers, nameplates, panels, and poles, and what research gaps and opportunities are identified in the literature?

The operation and inspection of electrical equipment are critical to maintaining the reliable operation of power systems [13]. Because equipment nameplates contain important textual information such as specifications, rated parameters, and manufacturing dates, collecting this information is necessary for the maintenance of such equipment [1]. However, during field inspections, nameplate images often present problems such as tilting, rotation, scale variations, and uneven illumination due to capture angles and installation positions, significantly affecting the performance of traditional recognition methods [4]. Furthermore, manual inspection is not only inefficient but also prone to human error [2], and despite the large volume of equipment image data collected during daily operations and maintenance, this data is not effectively utilized [3]. Therefore, accurate localization and recognition of text in equipment nameplate images are essential for the maintenance and reliable operation of electrical systems.

Beyond nameplates, recent literature shows that the problem extends to meters, LCD displays, substation cabinets, and other assets where technical information is presented in heterogeneous visual formats. In electrical meters, manual reading of meter values with multiple digits and symbols remains slow, costly, and error-prone, especially when real-time monitoring is required [14]. This need has driven a broad body of work on automatic meter reading, covering digital and energy meters [6,15,16,17,18,19], analog and pointer meters [20,21,22,23], and meter detection from UAV or substation imagery [24,25,26,27]. Similarly, in industrial LCD interfaces, characters may appear with low contrast, fine strokes, blur, deformation, scale variations, and complex backgrounds, which hinders localization prior to recognition [28].

At the international level, countries such as China, driven by continuous growth in electricity demand and large-scale energy infrastructure, have faced an urgent need to automate equipment inspection and maintenance processes. In this context, inspection robots have been developed to replace manual inspection, significantly increasing inspection efficiency and safety [29]. Nevertheless, many practical challenges remain; for example, the movement of inspection robots introduces vibrations that can blur captured images, compromising the quality of the captured data. Beyond robotic capture, a broad line of work targets substation operation and maintenance more generally. This includes the automatic acquisition of inspection data [30], the intelligent analysis of substation imagery with deep learning [31], machine vision condition monitoring of equipment [32], AI-based information extraction from substation engineering design [33], and equipment status diagnosis from infrared images combining target detection and information extraction [34].

In recent years, optical character recognition (OCR) technology has attracted considerable research attention for its contribution to the automation of daily information recording [35]. OCR technology generally involves two key steps: text detection and text recognition [10]. In practice, poor installation conditions and prolonged operation cause industrial meters to age to varying degrees, and during image capture, extreme lighting conditions, arbitrary viewing angles, and varying camera distances pose significant challenges for accurate OCR. To address these limitations, hybrid architectures such as Convolutional Recurrent Neural Networks (CRNNs) have gained relevance for their ability to recognize character sequences in digital meters [29]. In parallel, generative adversarial networks (GANs) have been applied to blurred image restoration prior to recognition, improving input quality in mobile robotic inspection scenarios [29].

Along the same lines, recent studies suggest that system robustness depends not only on the recognition model but also on the effective integration of all upstream stages of the computer vision workflow. Noise removal in visible images of electrical equipment, perspective correction, small component segmentation, and precise localization of text regions emerge as necessary conditions for OCR to function reliably in real-world scenarios [36,37]. Furthermore, the integration of visual information with textual or contextual asset data is emerging as a promising strategy to improve equipment identification when the image alone proves insufficient [38]. Along these lines, named-entity recognition over power-equipment text with BERT-BiLSTM-CRF [39], the combination of Transformer text embeddings with OCR for component classification [40], and the integration of computer vision with large language models for automated asset onboarding [41] point to multimodal and language-driven information extraction as an emerging trend.

A notable drawback of CNN-based computer vision approaches, however, is the high computational cost involved in training a model from scratch. Recent methods address this challenge by reusing pre-trained models, which attain strong performance after only modest additional training on domain-specific datasets [5]. Lighting conditions have a major impact on the images processed by CNN models during inspection. Image preprocessing is therefore employed to reduce noise generated during image acquisition and transmission, improve contrast, and resolve overexposure [42]. Enhanced images can increase the training dataset, improve the model’s generalization capability, and reduce the risk of overfitting. Taken together, these technical challenges highlight the need to continue developing robust solutions that integrate detection, recognition, and image preprocessing in real electrical environments, highlighting an important avenue for future research.

### 3.2. Results for Research Question 2

What computer vision and deep learning algorithms and architectures are currently used for text and digit extraction from electrical equipment?

In response to the identified challenges, the literature describes a diverse set of algorithms and architectures that address each stage of the recognition process. These systems typically follow a pipeline comprising image preprocessing, text-region detection, and text recognition [3]. The output of each stage serves as input to the next. The evolution of these pipelines has shifted from classical computer vision methods toward increasingly integrated deep learning architectures, driven primarily by the limitations of traditional approaches when faced with real-world inspection conditions. The following subsections discuss each stage of this pipeline in detail, progressing from traditional methods through text detection and text recognition to fully end-to-end systems.

#### 3.2.1. Traditional Methods

Classical approaches to text detection rely on manually designed visual features. Among the most representative are the Stroke Width Transform (SWT), which groups pixels with consistent stroke widths to form lines of text, and Maximally Stable Extremal Regions (MSER), which leverage the stability of certain regions under intensity transformations to isolate text candidates [2]. Similarly, sliding-window methods classify image regions as text or non-text using classifiers such as SVMs on texture features. Although these methods are computationally efficient, they have significant limitations: they are sensitive to scale changes, font variations, and multiple text orientations [2], conditions commonly encountered in industrial electrical inspection environments where equipment may be tilted, partially occluded, or exposed to uneven lighting [4].

The weaknesses of these methods lies in the assumptions embedded in their handcrafted features. SWT exploits the fact that text strokes tend to preserve relatively consistent widths; however, in electrical nameplate images, blur, surface degradation, engraving, or specular highlights can interrupt character contours and alter the apparent stroke width. MSER depends on stable intensity regions and sufficient contrast between characters and background, making it vulnerable to local overexposure, shadows, low-contrast plates, or reflections that split characters into fragments or merge them with the plate surface. Similarly, Hough transform- and edge-based procedures rely on reliable edge maps, clean geometric contours, and accurate prior localization; when meters or nameplates are captured at oblique angles, under reflections or occlusions, or with mixed font sizes and dense small text, these assumptions become difficult to satisfy. This explains why traditional pipelines tend to perform well in controlled settings but lose robustness when applied to field inspection images.

These limitations are also evident in studies of electrical equipment. Yang et al. identified approaches based on morphological image processing, segmentation, SVM, template matching, and manual feature extraction, but note that their performance degrades in the presence of complex backgrounds, noise, lighting variations, and typographic diversity [3]. Similarly, in reading analog meters, techniques such as the Hough transform, edge detection, or geometric segmentation are still used to estimate pointer angles and meter readings; however, these methods require precise prior localization of the dial and are sensitive to occlusions, reflections, or shadows from the pointer [43,44,45]. On electrical equipment nameplates, approaches based on traditional OCR pipelines show lower robustness against slanted text, mixed fonts, complex Chinese characters, or deteriorated backgrounds, which has driven the transition toward hybrid models combining deep-learning-based detection, neural text recognition, and post-OCR correction strategies [46,47].

#### 3.2.2. Text Detection

The adoption of convolutional neural networks (CNNs) marked a turning point in text detection. The first deep learning-based detectors, inspired by Faster R-CNN, operated with horizontal bounding boxes, which limited them to text aligned with the image axis [2]. This limitation was gradually overcome with architectures such as TextBoxes++, capable of regressing bounding boxes in multiple orientations, and methods based on bidirectional LSTM that generate high-density text proposals [2]. More recent scene-text detectors handle arbitrarily shaped text through intra- and inter-instance collaborative learning [48], while Transformer-based recognizers [49] and text detection and recognition by mobile robots in natural scenes [50] provide general-purpose approaches adaptable to electrical inspection tasks.

A particularly important development in electrical-equipment text detection is the Differentiable Binarization Network (DBNet), which incorporates differentiable binarization into the training process, eliminating the need for fixed thresholds in post-processing [10]. This feature makes it robust to blurred edges and uneven lighting, recurring issues in images captured in the field. Building on this foundation, recent work has proposed improved versions aimed at balancing accuracy and computational efficiency. On the one hand, lightweight feature extraction networks such as MobileOne and Feature Pyramid Network (FPN) modules with RepRes blocks have been incorporated for real-time applications [10,51]. On the other hand, although EBiDNet was developed for LCD interfaces in industrial control equipment rather than specifically for nameplates, it illustrates the potential of integrating EfficientNetV2-S and a Bidirectional Feature Pyramid Network (BiFPN) into the DBNet framework. Its bidirectional feature propagation enables a more balanced fusion of low-level textural details and high-level global semantic information. EBiDNet achieved an F1-score of 91.94% while using 17.96% fewer parameters than the original DBNet [28], suggesting that this architecture could be further explored for visually complex nameplate inspection tasks in the electrical domain. Specifically, for nameplates, other detectors enhance multiscale feature fusion for power-equipment nameplate text [52] or combine image enhancement with template matching in an end-to-end OCR scheme [53].

The YOLO family appears most frequently in tasks involving meters, cabinets, and regions of interest (ROIs). YOLOv5 is used for reading digital meters and industrial labels [11,36]; YOLOv7 is adapted for detecting irregular components in terminal blocks using YOLOv7-AO and YOLOv7-DBAH [37]; YOLOv8 and YOLOv8n are used for meter reading, terminal detection, and secondary wiring modeling [43,54,55]; and YOLOv11 appears in specialized microcharacter tasks for secondary wiring robots [12]. A common trend is to modify the base architecture with attention mechanisms, multiscale fusion, geometric losses, or lightweight modules to improve the detection of small targets while maintaining inference speed. These architectures have also been applied to the digitization of electrical schematics and wiring diagrams: YOLO combined with CRAFT and CRNN for substation one-line diagrams [56], YOLOv5 with PP-OCR for substation wiring diagrams [57], a Swin-Transformer-enhanced Faster R-CNN for single-line diagram symbols [58], and an optimized YOLO11 combined with CAD parsing for wiring-diagram recognition [59].

Other studies continue to investigate two-stage detectors such as Faster R-CNN or compare R-CNN and SSD, particularly for photovoltaic substation meters and nameplate recognition. Although these methods can improve accuracy in some scenarios, they typically involve higher computational costs and lower inference speeds than YOLO variants, making them more suitable for applications where localization accuracy takes priority over real-time performance [60,61,62].

For text detection under severe tilt conditions, approaches have been proposed that combine multidirectional Log-Gabor filters with ResNet-based adaptive multiscale fusion (ASF) modules, followed by differentiable segmentation to precisely delineate the nameplate region [4]. This architecture achieves recognition rates of 94.3% for tilted nameplates and 89.7% under strong lighting conditions, with a latency of 28.6 ms per frame [4].

#### 3.2.3. Text Recognition

Once text regions are detected, text recognition constitutes the second critical stage of the pipeline. In the inspection robot study by Lin et al., the CRNN (Convolutional Recurrent Neural Network) architecture is used as the core recognizer because it combines a CNN for visual feature extraction with an RNN for character sequence modeling and employs the CTC (Connectionist Temporal Classification) algorithm for final decoding. This architecture is particularly well-suited for digital LED meters, where digits form sequences of variable length. Additionally, GANs act as a preprocessing stage prior to CRNN recognition, enabling image restoration and character recognition to operate sequentially within the same pipeline.

Modern OCR engines are also playing an increasingly important role in electrical applications. PP-OCRv3 is used on contrast-enhanced plates and for detecting wiring in electrical cabinets, combining fine-grained text detection with subsequent recognition [11,63]. PP-OCRv5 is used in conjunction with 3D plates and asset records, demonstrating that lightweight OCR engines can be integrated with semantic techniques to link the recognized text to equipment inventories or master ledgers [64]. ViT-OCR is deployed in substation terminals as a number-recognition component within a pipeline that includes YOLOv8 and SegFormer [54].

For more complex contexts, Transformer-based architectures such as VIPTR integrate patch embeddings with pyramidal feature extraction and self-attention mechanisms, achieving richer visual and semantic representations than conventional CRNNs [1]. When dealing with text on electrical cabinet plates with a high diversity of designs, cloud-based OCR APIs have also been used in combination with synthetic data generated from 3D models. This approach mitigates the scarcity of labeled samples and achieved a recognition rate of 100% under controlled conditions [2]. In another study, CTPN is combined with a Transformer architecture to recognize text on electrical panels featuring multiple character types and slanted text [65]. These results are consistent with broader evidence from technical OCR, where TrOCR outperforms CRNN and Tesseract on degraded text, although its transfer to electrical panels requires domain-specific fine-tuning. In engineering document settings, generative data augmentation has further been used to fine-tune Transformer-based OCR models, mitigating the scarcity of labeled samples [66].

Beyond text recognition in isolation, the reviewed corpus shows a growing shift toward Vision Transformers and multimodal architectures for electrical equipment identification. Cui and Niu adopt a standard Vision Transformer (ViT) as the image feature extraction backbone within a multimodal fusion model for power equipment identification, combining it with a Transformer-based text encoder and a fused recognition stage [38]. Similarly, Zhang et al. propose a knowledge extraction method that aligns image and text records for power equipment operation and inspection, using a multimodal fusion module on top of a frozen alignment network [13]. These studies indicate that the adoption of self-attention-based visual backbones and multimodal fusion is already emerging within the electrical inspection domain, beyond their use inside OCR pipelines. However, the corpus reviewed here does not include foundation vision–language models (i.e., large-scale, pretrained VLMs such as those used for open-vocabulary visual question answering) applied specifically to electrical equipment inspection, which remains a promising research direction rather than an established practice in this domain.

#### 3.2.4. End-to-End Systems

A fundamental limitation of two-stage pipelines (separate detection and recognition) is the accumulation of errors: errors introduced during the detection stage inevitably propagate to the recognition stage [1]. End-to-end systems address this weakness by jointly optimizing both tasks using a unified loss function, allowing the recognition component to influence the feature representations learned by the detector.

**Figure 6:**
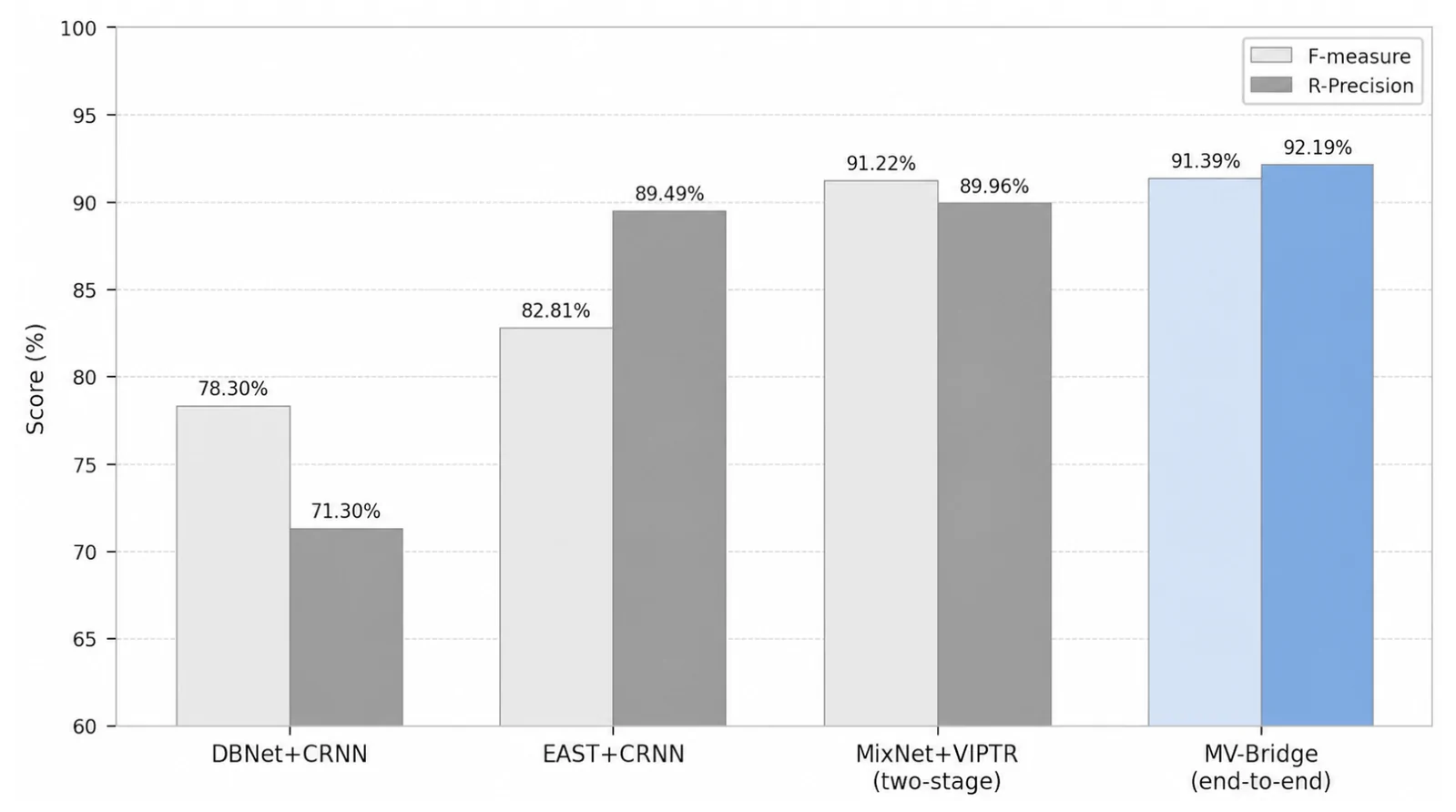
Performance comparison of MV-Bridge against classic two-stage pipelines on the RSEN dataset [1]. *Note: Blue bars highlight the end-to-end MV-Bridge method, while gray bars represent the classic two-stage pipelines*.

MV-Bridge is an advanced end-to-end approach for electrical equipment nameplates: it integrates MixNet as the detector and VIPTR as the recognizer via a Transformer-based Bridge module and lightweight Adapter modules, keeping the weights of both backbone models frozen to preserve pre-trained knowledge [1]. As shown in Figure 6, this joint training strategy allows MV-Bridge to outperform both classic two-stage pipelines and its direct predecessor MixNet+VIPTR on the publicly available https://gitee.com/laofeiwei/nameplate-datasets (accessed on 17 May 2026), achieving an F-measure of 91.39% and an R-Precision of 92.19% [1].

In a similar vein, the CTPN+Transformer system integrates sign localization/correction, text detection, and recognition using a Transformer architecture, although its modules are trained in stages [65]. A related nameplate-oriented pipeline is proposed by Liu et al., who combine preprocessing, CTPN-based text line detection, CRNN-CTC recognition, and Apache Flink-based parallelization to improve the efficiency of electrical equipment nameplate recognition under conditions involving multidirectional, noisy, blurred, or curved text [67].

In robotic inspection applications, the modular pipeline remains predominant due to their maintenance flexibility: Polygon-YOLOv5 detects and delineates the meter region while correcting perspective, YOLOv5s localizes the numerical regions, and CRNN performs the final digit recognition, achieving an overall accuracy of 98% [29]. For text recognition in electrical distribution networks, YOLOv8 combined with PaddleOCR achieves 100% accuracy in recognizing text labels under laboratory conditions [5].

In cabinets and secondary circuits, integrated systems tend to go beyond OCR. Cao et al. combine YOLOv7-AO, YOLOv7-DBAH, affine correction, and PaddleOCR to detect irregular components and recognize text on terminal blocks [37]. Li et al. integrate YOLOv5s-CBS and PP-OCRv3 to verify wiring in electrical cabinets [11]. Another study uses YOLOv8, SegFormer, and ViT-OCR to detect terminals, segment cables, and construct a digital model of the secondary circuit [54]. Song et al. propose a solution for wiring robots using YOLOv11, index-digit mapping, and SAST-CRNN for bent or occluded numbered conduits [12]. These studies show that, in substations, OCR is increasingly integrated with structural validation, topology, wiring rules, and digital modeling.

**Figure 7:**
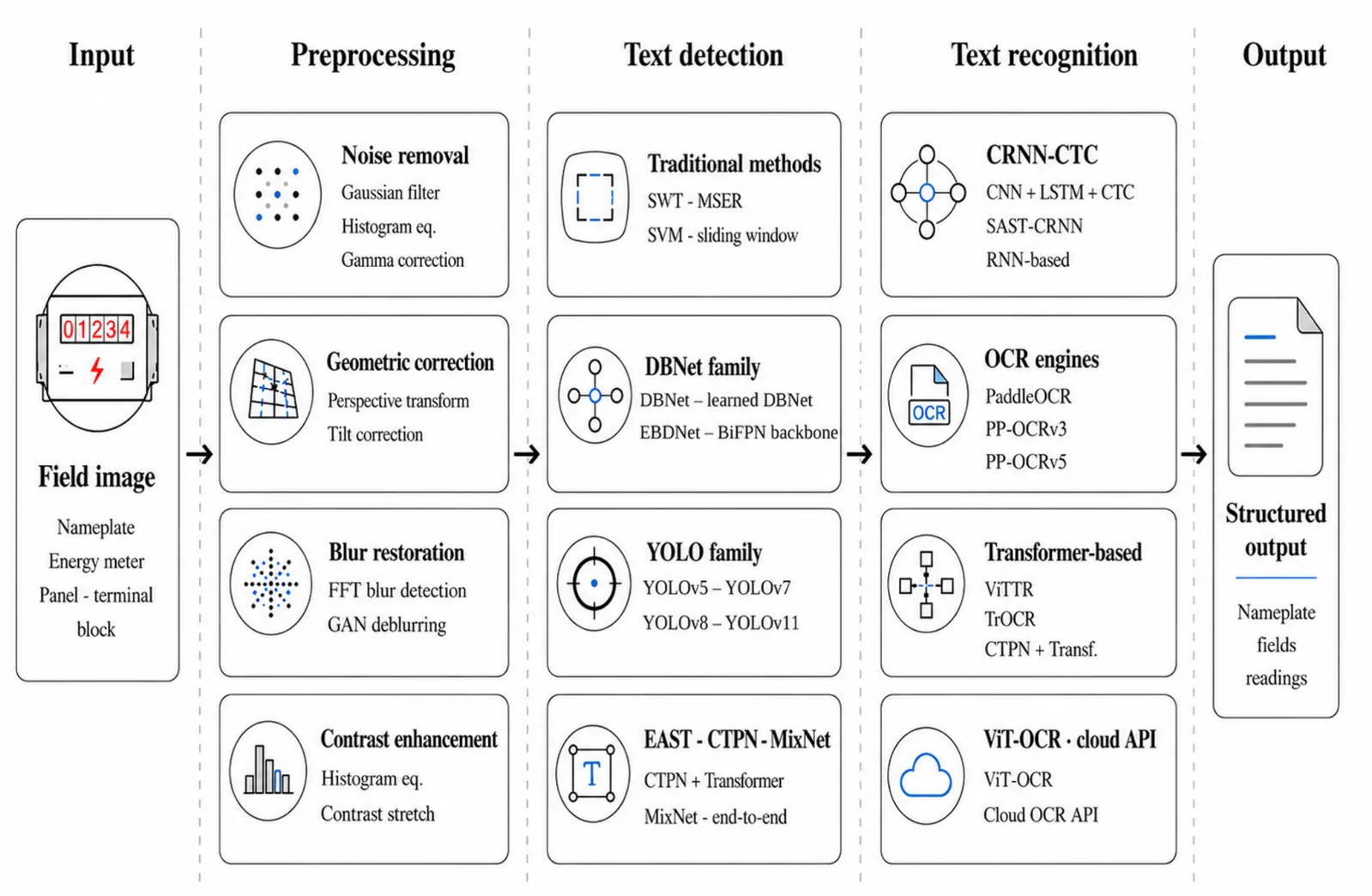
Computer vision pipeline for text extraction from electrical equipment. The preprocessing stage includes noise removal, geometric correction, blur restoration, and contrast enhancement techniques [4,29,36,63]. The text-detection stage summarizes traditional SWT-, MSER-, and SVM-based methods [2]; DBNet and related variants [10,28,51]; YOLO-based detectors [11,12,37,54,55]; and EAST-, CTPN-, and MixNet-based approaches [1,65,67]. The text-recognition stage comprises CRNN–CTC and related recurrent architectures [12,29]; PaddleOCR and PP-OCR engines [11,37,64]; Transformer-based recognizers such as VIPTR and TrOCR [1,66]; and ViT-OCR or cloud OCR services [2,54]. The final stage represents the conversion of the recognized information into structured nameplate fields and meter readings.

Taken together, the methods discussed above can be mapped onto the core stages of the recognition pipeline. Figure 7 synthesizes this landscape, organized by stage: image preprocessing, text detection spanning both classical feature-based methods and deep learning detectors, text recognition ranging from sequence-based to Transformer-based models, and fully end-to-end integration.

Across application scenarios, the distribution of architectures reflects the structure of the recognition problem rather than a uniform preference for a single model family. YOLO-based detectors are especially common in meter reading, terminal-block inspection, and cabinet analysis because these tasks often require fast localization of a limited set of objects or regions of interest under operational time constraints. In these settings, the main trade-off is between small-object detection accuracy and real-time inference, which explains the frequent use of lightweight backbones, attention modules, and multiscale feature fusion. By contrast, nameplate-oriented text spotting involves denser text fields, multi-line layouts, variable character sizes, and stronger dependence between detection and recognition. This favors DBNet, CRNN, Transformer-based recognizers, and end-to-end systems, which are better suited to modeling text structure and reducing error propagation across pipeline stages. Two-stage detectors remain useful when localization precision is prioritized, but their higher computational cost limits their suitability for embedded or robotic inspection. These differences also affect annotation cost: object-level labels may be sufficient for meters or terminal components, whereas nameplate text spotting often requires line-, word-, or character-level annotations, making dataset construction more expensive and reinforcing the need for synthetic data and transfer learning.

Table 8 consolidates the qualitative comparison discussed throughout this section, contrasting the main algorithmic families by typical use scenarios, main advantages, trade-offs, and deployment suitability.

### 3.3. Results for Research Question 3

What experimental environments, datasets, and evaluation metrics and methodologies are used to measure the performance of these approaches in recent literature?

#### 3.3.1. Experimental Environments

When reported, the reviewed studies described GPU-accelerated deep learning environments that combined workstation-class hardware with embedded devices for field deployment. Tan et al., for example, used an NVIDIA Jetson AGX Xavier and a GeForce RTX 2080 Ti GPU (NVIDIA Corporation, Santa Clara, CA, USA). Their software environment comprised Ubuntu 18.04, PyTorch 1.8, and CUDA 10.2 [4]. This configuration illustrates a hybrid experimental setting in which high-performance GPU resources support model development, while an embedded platform enables evaluation under deployment constraints.

Miao et al. provided a comparatively detailed description of their experimental environment for pointer-meter reading. The reported platform used Ubuntu 16.04.6 LTS, an NVIDIA TITAN XP GPU with 12,196 MiB of memory, CUDA 10.1, OpenCV 2.4.9, and the Paddle deep learning framework. However, the Paddle version was not specified. The trained method was subsequently deployed on an Atlas development board, although the exact board model and manufacturer were not identified in the source [44]. This study therefore provides both a training environment and an edge-deployment context, but does not report every detail required for complete replication.

Other studies provided only partial descriptions of their experimental settings. Lin et al. reported the dataset, evaluated models, and experimental results but did not specify Python, PyTorch, CUDA, Ubuntu, or GPU versions [29]. Li et al. described a pipeline based on YOLOv8, SegFormer, and ViT-OCR without providing the associated hardware, operating system, programming language, or framework versions [54]. Similarly, Song et al. reported the use of YOLOv11 and SAST-CRNN, together with a secondary wiring robot platform and field-acquired data, but did not provide a complete software or hardware configuration [12].

Overall, the available evidence does not support a general claim that a single programming language, deep learning framework, or hardware configuration predominates across the reviewed literature. The uneven level of reporting also limits reproducibility and direct comparison among studies. Accordingly, software versions and equipment details are stated here only when they were explicitly reported in the original publications.

In terms of image acquisition, studies employ images captured with mobile phones, UAVs, inspection robots, high-resolution cameras, or images of screens and nameplates taken under field conditions [12,29,37,54,60,68]. This heterogeneity confirms that the evaluation of these systems cannot be limited to controlled datasets, since final performance depends on lighting, angle, blur, distance, contrast, scale, and occlusion.

#### 3.3.2. Datasets

A common characteristic in the literature is the scarcity of standardized public datasets for electrical equipment nameplates, which leads most studies to build their own datasets or combine public data with their own collections. Reported dataset sizes range from small datasets of approximately 1000 images [29] to more robust collections exceeding 16,000 images [1].

For electrical equipment nameplates, Zhong et al. evaluate on 1200 nameplate images and complement with public scene text datasets such as ICDAR2015, SVT, and KAIST [47]. Zhang et al. train an RNN model with more than 9300 electrical equipment nameplate images obtained through direct image capture and online image searches, but evaluate the model on only 30 images [68]. In text spotting, Qiu et al. report a proprietary dataset of 550 nameplate images and a public dataset of 3949 text instances [65]. For analog meters, Chen et al. use 2133 training images, 533 test images, and 17,225 digit images for scale recognition [43].

The most frequently used public dataset is the https://gitee.com/laofeiwei/nameplate-datasets (accessed on 17 May 2026) which contains 14,934 training images and 1572 test images annotated in the ICDAR-2017 format, covering a wide variety of text, backgrounds, and spatial layouts [1]. Studies oriented toward real inspection conditions incorporate controlled variability in their own collections, including different tilt angles (0°–60°), illumination levels, scales, and partial occlusions [4]. A particular case is electrical cubicle systems, where the high variability of designs and the scarcity of samples per type led researchers to generate synthetic data from 3D models, a strategy that reduces labeling costs and computational time [5]. For the restoration of blurred images captured by inspection robots, the GoPro dataset was used, comprising 3214 pairs of blurred and sharp images [29].

#### 3.3.3. Evaluation Metrics and Methodologies

Performance evaluation in the reviewed systems decomposes into two levels: detection quality and recognition quality. For text detection, the standard metrics are Precision (P), Recall (R), and F-measure (F), consistently employed in works oriented toward text localization in nameplates [1,10]. Some studies extend this set with specialized indicators such as NAED (Normalized Average Edit Distance) and R-Precision to capture character-level recognition errors [1], or with custom indicators such as missed detection rate, recognition rate, and skew angle deviation [35]. In object detection tasks applied to meters and cabinets, mAP (mean average precision) at different IoU thresholds is the dominant metric [11,54].

For the evaluation of image preprocessing stages, PSNR (Peak Signal-to-Noise Ratio) and SSIM (Structural Similarity Index) predominate, used to quantify the quality of restored or enhanced images [29,42]. PSNR quantifies reconstruction fidelity, with higher values indicating lower distortion; SSIM measures structural similarity based on luminance, contrast, and structure, with values close to 1 indicating greater fidelity with respect to the original image. In systems under adverse lighting conditions, these are complemented with operational metrics such as precision under strong light and latency per frame [4], reflecting the importance of real-time performance for industrial inspection applications. In meter reading, metrics further adapt to the nature of the task: relative reading error and tolerance-based precision replace detection-oriented scores, given that the output of interest is a numeric value rather than a bounding box or a character sequence [43,45,62].

**Figure 8:**
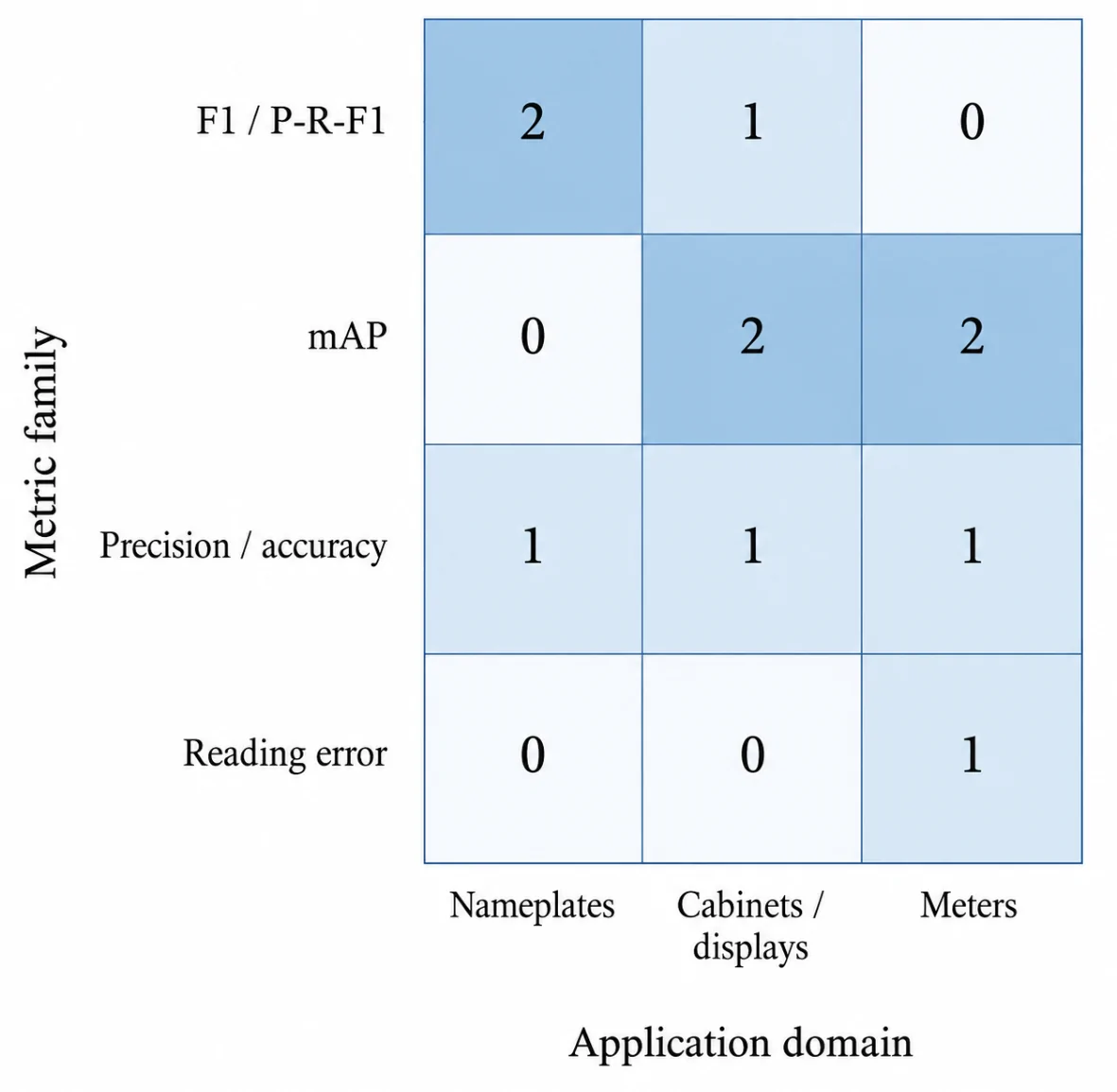
Fragmentation of metric families across application domains. Cell values indicate the number of studies for each metric–domain combination. The visualization highlights fragmented evaluation practices and should not be interpreted as a direct performance ranking.

Because the reviewed studies use different tasks, datasets, and evaluation protocols, the reported values should be read as descriptive evidence rather than as a unified benchmark. Figure 8 therefore provides a compact overview of how the different metric families are used across the main application domains before the detailed results are presented in Table 9.

Table 9 summarizes the metrics and reported results across the reviewed studies, organized by application domain. Each row corresponds to an independent study conducted using a different dataset, under different experimental conditions, and evaluated with metrics selected by the authors according to their specific objectives. To improve readability, the table uses a consistent notation: P denotes precision, R denotes recall, F1 denotes the harmonic mean between P and R, Hmean is reported as F1 (Hmean) when that terminology is used in text-detection studies, mAP denotes mean average precision, and mAP@0.5 denotes mAP computed at an IoU threshold of 0.5. Acc. denotes accuracy, and R-Prec. denotes R-Precision.

The summary in Figure 8 and the detailed entries in Table 9 show that the reviewed studies report results on heterogeneous datasets using incompatible metrics, which prevents any direct quantitative comparison across methods or application domains. Detection-oriented works favor P/R/F1 or mAP-based metrics; recognition works use accuracy or R-Precision; and meter reading adopts task-specific error indicators. This fragmentation is not incidental: it reflects the absence of a unified benchmark for electrical equipment text extraction, which is one of the central gaps identified in the literature. Consequently, the analysis presented here is necessarily qualitative in nature, focusing on methodological trends, dominant architectural choices, and evaluation practices rather than on a quantitative ranking of methods.

## 4. Limitations

The present review has several limitations that should be considered when interpreting its findings. The most significant limitation lies in the reviewed literature itself: the near-total absence of standardized, publicly available benchmarks forces most studies to rely on small, custom-built datasets of a few hundred to a few thousand images, often collected under a single set of acquisition conditions, which limits the generalizability of the reported results to broader real-world deployment. Second, the heterogeneity of evaluation metrics across studies—detection-oriented P/R/F1, mAP-based object detection scores, recognition accuracy, and task-specific reading error—prevented a quantitative meta-analysis and restricted the synthesis presented here to a qualitative comparison, limiting the ability to rank methods objectively. Third, the formal search was restricted to five major scientific sources and two exploratory search engines; therefore, relevant regional or highly specialized sources may have been missed. Finally, many studies report performance under controlled or semi-controlled conditions and do not systematically evaluate robustness against the adverse conditions identified in Section 3 (tilt, occlusion, blur, or extreme illumination), so that the reported metrics may overstate real-world performance.

## 5. Conclusions and Future Work

The findings of this review confirm that text recognition on electrical equipment nameplates remains an open technical problem. Among the main difficulties identified, two stand out and are closely related: the gap between performance reported under controlled conditions and the demands of real inspection environments, and the absence of standardized public benchmarks for the electrical domain, which together prevent objective comparison between methods and limit the accumulation of knowledge in the field. The transition from traditional methods toward deep learning architectures is nonetheless well established, with DBNet and CRNN among the most frequently used approaches and end-to-end systems representing a prominent research direction.

Based on the evidence synthesized in this review, we recommend that future methodological developments in this domain converge toward end-to-end, lightweight architectures trained with domain-specific pretraining and fine-tuning, following the trajectory already observed from two-stage pipelines (e.g., DBNet+CRNN) toward integrated systems (e.g., MV-Bridge). This path should be pursued jointly with the development of standardized, publicly available benchmark datasets that span nameplates, meters, and cabinets under real-world conditions and define shared evaluation protocols. Prioritizing this combination—integrated architectures evaluated on shared, realistic benchmarks—offers the most direct methodological path forward for the field.

This review provides a basis for more focused reviews of individual subdomains. Future work should expand the search by incorporating additional databases and search terms in languages other than English, given that a significant share of scientific output in this field originates from non-English-speaking contexts.

Future work should primarily address the data limitations identified throughout this review. The construction of public datasets reflecting real electrical inspection conditions remains a foundational need, and early work already points toward practical ways to approach it: synthetic data generation, through SynthText-style rendering and GAN-based sample synthesis, has been used to mitigate the scarcity of labeled samples in cabinet and nameplate recognition [2,5], while transfer learning and weakly supervised approaches offer a complementary path to reduce dependence on large annotated datasets and to improve domain adaptation across asset types and acquisition conditions [4,36]. In this domain, dataset design should not only increase sample volume but also reproduce the visual and semantic difficulties of electrical assets: metallic or laminated plates that generate glare and local overexposure, dense small text arranged in multi-line technical layouts, mixed fonts or engraved characters, and parameter fields whose values must be interpreted together with units, serial numbers, and equipment-specific terminology. Future datasets should also support explicit testing of cross-domain generalization, for example between transformer nameplate, meter, cabinet, and field-capture scenarios, so that adaptation strategies can be evaluated beyond a single acquisition setting.

Accordingly, visual recognition should be complemented with domain-aware validation rather than relying on OCR confidence alone. On the deployment side, closing the gap between laboratory and field performance will likely depend on lightweight edge AI architectures deployable on embedded devices, building on early efforts already reported in the literature [4,44]. At the same time, federated or privacy-preserving learning could enable utilities and infrastructure operators to improve models collaboratively without centralizing sensitive inspection images. As the field matures, explainable deep learning, through prototype-based architectures already proposed for OCR tasks, would support the interpretability required for industrial deployment [2]. Multimodal approaches that integrate visual information with textual maintenance records also offer a promising way to enrich context-aware inspection systems [13,38]. These directions—domain adaptation, synthetic data generation, edge AI deployment, explainable AI, federated learning, and standardized benchmarks—define a more concrete research agenda for moving from isolated prototypes toward robust, comparable, and deployable inspection systems.

## Figures and Tables

**Table 1:** Keywords used in the search.

Group	Approach	Keywords
Group 1	Application domain	“power equipment”, “electrical transformers”, “energy meters”, “electrical panels”, “utility assets”, “substation”, “switchgear”, “circuit breaker”, “distribution box”, “nameplate”
Group 2	Text extraction task	“text extraction”, “digit recognition”, “OCR”, “text detection”, “character recognition”, “meter reading”, “label recognition”, “serial number recognition”
Group 3	Computer vision and OCR technologies	“computer vision”, “deep learning”, “machine learning”, “YOLO”, “EAST”, “CRNN”, “Tesseract”, “PaddleOCR”, “CNN”, “RNN”, “Mask R-CNN”, “SSD”, “MobileNet”, “DBNet”, “TrOCR”
Group 4	Field conditions and evaluation metrics	“harsh environment”, “real-world conditions”, “low illumination”, “occlusion”, “rust”, “corrosion”, “accuracy”, “precision”, “recall”, “F1-score”, “mAP”, “CER”, “WER”, “performance evaluation”

**Table 2:** Search strings used.

Combination of Groups	Search String
Group 1 + Group 2	(“power equipment” OR “electrical transformers” OR “energy meters” OR “electrical panels” OR “utility assets” OR “substation” OR “switchgear” OR “circuit breaker” OR “distribution box” OR “nameplate”) AND (“text extraction” OR “digit recognition” OR “OCR” OR “text detection” OR “character recognition” OR “meter reading” OR “label recognition” OR “serial number recognition”)
Group 1 + Group 3	(“power equipment” OR “electrical transformers” OR “energy meters” OR “electrical panels” OR “utility assets” OR “substation” OR “switchgear” OR “circuit breaker” OR “distribution box” OR “nameplate”) AND (“computer vision” OR “deep learning” OR “machine learning” OR “YOLO” OR “EAST” OR “CRNN” OR “Tesseract” OR “PaddleOCR” OR “CNN” OR “RNN” OR “Mask R-CNN” OR “SSD” OR “MobileNet” OR “DBNet” OR “TrOCR”)
Group 1 + Group 2 + Group 3	(“power equipment” OR “electrical transformers” OR “energy meters” OR “electrical panels” OR “utility assets” OR “substation” OR “switchgear” OR “circuit breaker” OR “distribution box” OR “nameplate”) AND (“text extraction” OR “digit recognition” OR “OCR” OR “text detection” OR “character recognition” OR “meter reading” OR “label recognition” OR “serial number recognition”) AND (“computer vision” OR “deep learning” OR “machine learning” OR “YOLO” OR “EAST” OR “CRNN” OR “Tesseract” OR “PaddleOCR” OR “CNN” OR “RNN” OR “Mask R-CNN” OR “SSD” OR “MobileNet” OR “DBNet” OR “TrOCR”)
Group 1 + Group 2 + Group 3 + Group 4	(“power equipment” OR “electrical transformers” OR “energy meters” OR “electrical panels” OR “utility assets” OR “substation” OR “switchgear” OR “circuit breaker” OR “distribution box” OR “nameplate”) AND (“text extraction” OR “digit recognition” OR “OCR” OR “text detection” OR “character recognition” OR “meter reading” OR “label recognition” OR “serial number recognition”) AND (“computer vision” OR “deep learning” OR “machine learning” OR “YOLO” OR “EAST” OR “CRNN” OR “Tesseract” OR “PaddleOCR” OR “CNN” OR “RNN” OR “Mask R-CNN” OR “SSD” OR “MobileNet” OR “DBNet” OR “TrOCR”) AND (“harsh environment” OR “real-world condition” OR “low illumination” OR “occlusion” OR “rust” OR “corrosion” OR “accuracy” OR “precision” OR “recall” OR “F1-score” OR “mAP” OR “CER” OR “WER” OR “performance evaluation”)

**Table 3:** Formal and exploratory information sources used in the search phase.

Data Source	Type	Date Last Searched	URL
IEEE Xplore	Digital library	23 May 2026	https://ieeexplore.ieee.org
Scopus	Database	23 May 2026	https://www.scopus.com
ACM Digital Library	Digital library	24 May 2026	https://dl.acm.org
ScienceDirect	Database	24 May 2026	https://www.sciencedirect.com
SpringerLink	Digital library	24 May 2026	https://link.springer.com
Google Scholar	Exploratory academic search engine	17 May 2026	https://scholar.google.com
Semantic Scholar	Exploratory AI-assisted academic search engine	17 May 2026	https://www.semanticscholar.org/

**Table 4:** Eligibility criteria applied during study selection.

Inclusion Criteria	Exclusion Criteria
Articles published in journals, conference proceedings, academic digital libraries, or recognized scientific repositories.	Unverifiable gray literature or documents lacking minimal bibliographic traceability.
Documents published in English between 2020 and 2026.	Documents published outside the defined time frame or in languages other than English.
Studies addressing the extraction, detection, or recognition of text, characters, or digits in electrical equipment, meters, nameplates, panels, substations, or other energy assets.	Studies on general OCR, document recognition, or computer vision without explicit application to the electrical domain or to comparable industrial contexts.
Studies that describe, evaluate, compare, or review computational algorithms, architectures, or methodologies for text and digit extraction and recognition.	Studies that do not present computational methods, experimental results, or relevant reviews that can contribute evidence to the research questions.
Studies that contributed evidence relevant to at least one research question.	Duplicate documents, documents without an accessible abstract or full text, or studies that do not provide direct evidence for the review.

**Table 5:** Evidence tiers used to group the included studies for synthesis.

Evidence Tier	Scope and Representative Targets
Direct evidence	Studies on text, character, or digit extraction from electrical equipment, including transformer and equipment nameplates, energy meters, and substation assets.
Adjacent industrial evidence	Studies on closely related industrial targets that present similar visual and technical challenges: industrial LCD interfaces, control cubicles and cabinets, terminal blocks, and electrical schematics or wiring diagrams.
Methodological (transferable) evidence	Methodological computer vision and OCR studies that are not specific to the electrical domain but whose detection or recognition methods are directly transferable, such as arbitrary-shaped scene text detection and transformer-based recognition.

**Table 6:** Initial search results from the five formal sources and the exploratory Semantic Scholar engine.

Data Source	Source Type	Q1	Q2	Q3	Q4	Total Results
IEEE Xplore	Digital library	144	2280	57	51	2532
Scopus	Bibliographic database	301	3033	81	60	3475
ScienceDirect ^*a*^	Digital library	382	2191	21	3	2597
SpringerLink	Digital library	431	5572	220	198	6421
ACM Digital Library	Digital library	313	1270	266	261	2110
**Formal sources subtotal**		**1571**	**14,346**	**645**	**573**	**17,135**
Semantic Scholar	Exploratory AI-assisted academic search engine	375	2138	64	48	2625
**Total initial records**	Formal sources + Semantic Scholar	**1946**	**16,484**	**709**	**621**	**19,760**

*Note: Boldface identifies the subtotal and overall total rows.* ^*a*^ ScienceDirect searches used reduced query strings because of the platform limit of eight terms or expressions per search.

**Table 7:** Adapted ScienceDirect search strings used during the search process.

Query	Adapted ScienceDirect Search String
Q1: Group 1 + Group 2	(“power equipment” OR “electrical transformer” OR “energy meter” OR nameplate) AND (OCR OR “text extraction” OR “text detection” OR “meter reading”)
Q2: Group 1 + Group 3	(“power equipment” OR “electrical transformer” OR “energy meter” OR nameplate) AND (“computer vision” OR “deep learning” OR YOLO OR TrOCR)
Q3: Group 1 + Group 2 + Group 3	(“power equipment” OR ”energy meter” OR nameplate) AND (OCR OR “text detection”) AND (“computer vision” OR “deep learning” OR YOLO)
Q4: Group 1 + Group 2 + Group 3 + Group 4	(“power equipment” OR nameplate) AND (OCR OR “text detection”) AND (“computer vision” OR “deep learning”) AND (“low illumination” OR accuracy)

**Table 8:** Qualitative comparison of algorithmic families for text and digit extraction on electrical equipment.

Family	Typical Use Scenario	Main Advantage	Main Trade-Off	Deployment Fit
Traditional methods (SWT, MSER, Hough, classical OCR)	Controlled OCR or geometric meter-reading pipelines	Very low computational cost; no training required	Sensitive to scale, orientation, lighting variation, reflections, and mixed fonts [2,4]	Suitable mainly in controlled settings
DBNet and variants	Text detection in nameplates, cabinets, and industrial displays	Differentiable binarization improves robustness to blurred edges and uneven lighting; EBiDNet reaches F1 = 91.94% with 17.96% fewer parameters [28]	Requires domain-specific fine-tuning and text-region annotations	Medium–high; lightweight variants support real-time use [10,51]
YOLO family	Meter reading, terminal blocks, cabinets, and region-of-interest detection	Fast localization; adaptable with attention and multiscale modules; YOLOv7-AO reaches 74.3 FPS at mAP@0.5:0.95 = 0.809 [37]	Small or densely packed objects may require architectural modifications	High; oriented toward real-time inspection
Two-stage detectors (Faster R-CNN, SSD)	Precision-oriented localization in meters, substations, or constrained targets	Higher localization precision	Higher computational cost and lower speed [60,61,62]	Limited for real-time use; useful when precision is prioritized
CRNN/modern OCR engines (PP-OCR, ViT-OCR)	Recognition of cropped text regions, labels, and digital meter values	Good precision–speed balance; CRNN with preprocessing raises accuracy from 86.4% to 98.8% [29]	Sensitive to detection quality and degraded inputs	High when preprocessing and localization are stable
Transformer-based recognition (VIPTR, TrOCR)	Complex nameplate text spotting, degraded text, and multi-line layouts	Richer visual and semantic perception than CRNN	Higher training cost and stronger data requirements	Medium; constrained by edge-device resources
End-to-end systems (MV-Bridge, CTPN+Transformer)	Integrated nameplate text spotting and detection–recognition pipelines	Reduces error propagation between detection and recognition; MV-Bridge reaches F1 = 91.39% on RSEN [1]	Higher joint-training and integration complexity	Medium; heavier for edge deployment

**Table 9:** Reported performance results by application domain.

Method	Dataset	Metric	Reported Value
Nameplates			
Improved DBNet [51]	Power nameplate, custom	F1 (Hmean)/P/R	91.3%/92.2%/90.3%
EBiDNet [28]	LCD interface, custom	F1	91.94%
RNN [68]	Power nameplate, custom	P	99.30% (alphanumeric)
MV-Bridge [1]	RSEN (public)	P/R/F1	90.86%/91.93%/91.39%
Cabinets and terminal strips			
YOLOv5s-CBS + PP-OCRv3 [11]	Wiring labels, custom	mAP@0.5/P/R	96.0%/96.0%/91.7%
YOLOv8 [54]	Substation terminals, custom	mAP@0.5	98.3%
YOLOv8 + SegFormer + ViT-OCR [54]	Substation terminals, custom	OCR Acc.	97.6%
Meter reading			
Image enhancement + YOLOv8 [45]	Pointer meters, custom	Avg. reading error	0.37%
Multi-class pointer meter [43]	Pointer meters, custom	P (±5% tolerance)	86.43%
Faster R-CNN X101-FPN [62]	Photovoltaic substation	mAP	60.38
Faster R-CNN R101-FPN [62]	Photovoltaic substation	mAP	58.99

## Data Availability

No new data were created or analyzed in this study. Data sharing is not applicable to this article.
